# Mechanisms of Cynarine for treatment of non-alcoholic fatty liver disease based on the integration of network pharmacology, molecular docking and cell experiment

**DOI:** 10.1186/s41065-022-00256-7

**Published:** 2022-12-01

**Authors:** Chun-Yong Sun, Le-Le Yang, Pan Zhao, Pei-Zheng Yan, Jia Li, Dong-Sheng Zhao

**Affiliations:** 1 College of Pharmacy, Shandong University of Traditional Chinese Medicine, No. 4655 Daxue Road, Jinan, 250355 China; 2 State Key Laboratory of Quality Research in Chinese Medicine, Institute of Chinese Medical Sciences, University of Macau, Macau, 999078 China

**Keywords:** Cynarine, Non-alcoholic fatty liver disease, Network pharmacology, Integration analysis, Molecular docking

## Abstract

**Background:**

Nonalcoholic Fatty Liver Disease (NAFLD) is a chronic Liver Disease prevalent all over the world. It has become more and more common in Japan, China and most western developed countries. The global prevalence rate is 25.24%, and the trend is increasing year by year. Related studies have shown that Cynarine has certain liver protection, lipid lowering and immune intervention effects. So, this study to systematically predict and analyze the mechanism of Cynarine in the treatment of non-alcoholic fatty liver disease (NAFLD) based on the integration of network pharmacology, molecular docking, and cell experiment.

**Methods:**

We performed Heatmap and Venn diagram analyses to identify genes and targets in Cynarine treat NAFLD. The network of Cynarine-therapeutic targets and the protein-protein interaction network (PPI) was constructed. We used gene ontology (GO) and Kyoto Encyclopedia of Genes and Genomes (KEGG) enrichment analyses to visualize associated functional pathways. The Sybyl tool was used to dock the Cynarine with key therapeutic targets molecularly. Finally, cell experiments were applied to validate the role of Cynarine in the treatment of NAFLD.

**Results:**

The Cynarine could act on 48 targets of NAFLD, and the role of CASP3, TP53, MMP9, ELANE, NOTCH1 were more important. The PPI network showed that immune and inflammation-related targets played a pivotal role. The KEGG analysis found that the PI3K-Akt signaling pathway, cell cycle and MAPK signaling pathway may be the main pathways for Cynarine to prevent and treat NAFLD. Molecular docking studies confirmed that Cynarine has good binding activity with therapeutic targets. Cynarine reduced the fat deposition ability of NAFLD model cells, and effectively reduced the levels of ALT and AST released by liver cells due to excessive lipid accumulation. We also found that Cynarine inhibited the expression of AKT1 and MAPK1.

**Conclusions:**

This study revealed that Cynarine could significantly reduce the fat deposition ability of NAFLD model cells, which may be closely related to the effective regulation of AKT1 and MAPK1 expression by Cynarine.

**Supplementary Information:**

The online version contains supplementary material available at 10.1186/s41065-022-00256-7.

## Introduction

Non-alcoholic fatty liver disease (NAFLD) is one of the most common chronic liver diseases, which affects approximately 25% of adults worldwide. It is characterized by fatty degeneration of liver parenchymal cells with the absence of heavy drinking and no other clear liver damage factors [1]. In recent years, the prevalence of NAFLD in China has been increasing and with a trend of patients getting younger. Studies have shown that the prevalence of NAFLD in urban areas and rural areas in China is 21.83 and 20.43% [2]. At first, NAFLD was considered to be a kind of benign disease. With the continuous in-depth study of the disease, it has been discovered that NAFLD will possibly develop into liver fibrosis and is closely related to end-stage liver diseases such as liver cirrhosis, liver cancer, and liver failure, which poses a serious threat to public health [3, 4]. At present, it is not very clear about the pathogenesis of NAFLD, and its treatment measures are mainly to change bad living habits and control metabolic factors [1]. A single treatment method is difficult to achieve the desired effect. However, blindly multi-drug intervention may increase the burden on the liver. Therefore, looking for an effective and diversified approach to prevention and treatment is of great significance for treating NAFLD and could reduce the incidence of end-stage liver disease caused by it. Based on dialectical treatment, the Traditional Chinese Medicine (TCM) compound that has been clinically verified for a long time and has the advantages of multiple action pathways is undoubtedly worthy of further research.

Cynarine is one of the major caffeoylquinic acid compounds extracted from the TCM artichoke (*Cynara scolymus* L.), which has anti-obesity and anti-oxidant liver effects in high fat diet-induced obese rats [5, 6]. It has also been reported that Artichoke leaf extract has liver-protective effects and causes downregulation of oxidative stress in acute diazinon-induced liver injury in rats [7]. Therefore, it is concluded that Cynarine may play a role in treating NAFLD.

However, the current research is mostly limited to a certain target or a certain pathway to discuss the action mechanism of Cynarine. The characteristics of its multi-target and multi-pathway couldn’t be reflected, which failed to explain the mechanism of Cynarine in protecting liver. Network pharmacology combines multi-disciplinary research methods and content, including hierarchical network construction and analysis, bioinformatics, computational biology, and multi-directional pharmacological analysis. It can transform from a single target and single approach research form to a holistic and systematic form on exploring the relationship between drugs, targets, and diseases, consistent with the overall view of Chinese Medicine [8].

In this study, a method of the integration of network pharmacology, molecular docking and cell experiments was used to discover cynarine’s multiple targets and multiple pathways for the treatment of NAFLD, which provided a scientific basis and research direction for the systematic development of Cynarine.

## Materials and methods

### Screening of potential targets of Cynarine

We searched the 3D structure of Cynarine (CAS: 30964-13-7) in the PubChem database (https://pubchem.ncbi.nlm.nih.gov/) [9] and obtained its SDF file, schema and PubChem CID. Then the 3D structure of Cynarine was input into PharmMapper (http://www.lilab-ecust.cn/pharmmapper/) [10] to predict its potential targets of Cynarine with the maximum return parameter. Also, SEA (http://sea.bkslab.org/) [11] was used in which results were limited to “*Homo sapiens*”. What’s more, we searched potential targets of Cynarine in SwissTargetPrediction (http://www.swisstargetprediction.ch/) [12] and targets with Probability> 0.10 were screened for subsequent analysis.

### Acquisition of target genes correlated with NAFLD

First, we analyzed differentially expressed genes (DEGs) for GSE89632 in the GEO database (https://www.ncbi.nlm.nih.gov/geo/), and DEGs (|logFC| ≥ 1 and *P-*value< 0.05) were selected for subsequent analysis. Then, we searched the “nonalcoholic fatty liver disease” keyword in three databases to get a comprehensive collection of NAFLD-related genes: targets with Relevance score > 10 in Genecards (https://www.genecards.org/), all targets in OMIM (https://omim.org/), and targets with Direct Evidence as marker/mechanism or therapeutic in CTD (http://ctdbase.org/) [13].

### Protein-protein interaction (PPI) network construction and its topological analysis

The Draw Venny Diagram online program (http://bioinformatics.psb.ugent.be/webtools/Venn/) was used to intersect the NAFLD-related targets and Cynarine target genes to obtain the common targets which were considered potential targets for Cynarine against NAFLD. Then we submitted potential targets to the String website (https://string-db.org/) [14] to generate a PPI network diagram with the minimum required interaction score as the default value and medium confidence as 0.400.

Also, we constructed PPI networks of Cynarine targets and NAFLD-related genes by BisoGenet (http://bio.cigb.edu.cu/bisogenet-cytoscape/) [15]. Then we performed network topology analysis in CytoNCA [16] to calculate PPI network topology parameters. The secondary network was filtered with DC > 74, and the core network was filtered with BC > 600.

### Cynarine -targets network construction

We input Cynarine, its candidate targets, and NAFLD to Cytoscape 3.7.1 software to construct the Cynarine - candidate target - NAFLD network by which we could predict the main targets of Cynarine in treating NAFLD.

### Gene Ontology (GO) and Kyoto Encyclopedia of Genes and Genomes (KEGG) pathway enrichment analysis

Firstly, we used R3.6.0 software and installed the RSQLite package and org.Hs.eg.db package to convert the potential gene name to gene ID. Then we installed colourspace, string, DOSE, cluster Profiler, path view and other program packages to conduct GO (Gene Ontology) and KEGG (Kyoto Encyclopedia of Genes and Genomes) pathways enrichment analysis. Both GO and KEGG enrichment analysis was statistically significant, with *P* < 0.05.

### Target-pathway network construction

We used Cytoscape 3.7.1 software to construct the gene-pathway network based on the top 30 enriched KEGG pathways and genes associated with these pathways.

### Molecular docking between Cynarine and targets

We used Sybyl × 2.1.1 for surfex-dock (semi-flexible docking mode). The top ten proteins were identified as receptors and Cynarine molecules as ligands. And we obtained the points of the ten proteins based on total score Function. The GSK3B protein (PDB ID: 4J1R) with the highest score was selected to map Cynarine using pymol.

### Non-alcoholic fatty liver cell model construction and drug treatment

In this study, a complete medium containing 200 μmol·L ^− 1^ sodium oleate was used to culture hepatocytes to establish a non-alcoholic fatty liver cell model. HepG2 cells in the logarithmic growth phase were inoculated and cultured in a 12-well plate at 10^4^/well. The normal medium was set as the control group; the medium containing 200 μmol·L ^− 1^ sodium oleate was used as the model group; 5 mg·L ^− 1^ Cynarine pre-treated the cells for 1 h [17], and then directly added 200 μmol·L ^− 1^ sodium oleate without washing to continue culturing the cells to make a NAFLD cell model. Each group had three repetitions.

### Oil red O staining

Take a small number of cells from each group, discard the cell culture medium, and wash with PBS; fix with 10% formaldehyde for 10 minutes, and put the oil red O staining solution in a 60-65 °C incubator to preheat; use Rinse the cells twice with PBS, add the preheated oil red O staining solution, and place in a 60-65 °C incubator for 15 min; 80% propylene glycol differentiates for 2 to 5 min, and observe under the microscope until the background is close to colorless; Rinse the cells three times with PBS, microscopically inspect, take pictures and record. Collect about 1.0 × 10^4^ cells in each group separately for 48 h, wash the cell surface oil red O with PBS; add 200 μL of isopropanol to extract the oil red O in the cells, centrifuge to collect the supernatant, and then The OD value was measured at a wavelength of 510 nm. The OD value of each group represents the quantitative value of fat accumulated by the cells of the group and is statistically analyzed.

### Biochemical testing

After the cells were cultured for 48 h, the culture supernatants of the cells of each group were collected and tested by an automatic biochemical analyzer for alanine transaminase (ALT) and aspartate transaminase (AST).

### qRT-PCR detection the expression levels of AKT1 and MPK1 in each group of cells

The trizol method was used to extract the total RNA of the three groups of cells from the control group, the model group and the drug treatment group and perform reverse transcription. Using the cDNA obtained by reverse transcription as a template, qRT-PCR was performed to detect the presence of AKT1 and MPK1 in each group of cells. At the same time, GAPDH was used as the internal reference gene, and the experiment was repeated three times for each sample.

### Western blot detection of AKT1 and MPK1 protein expression

Wash the three groups of cells cultured in a 6-well plate twice with PBS, add pre-cooled cell lysate for 30 min, 12,000 r/min, centrifugal radius 10 cm, centrifuge at 4 °C for 15 min, sediment cell debris Wait for impurities, take the supernatant, and determine the amount of protein by BCA method. Separate the total protein with 12% SDS-polyacrylamide gel electrophoresis, then transfer to polyvinylidene fluoride (PVDF) membrane, block with 5% BSA for 90 min, add the diluted primary antibody, and incubate at 4 °C overnight. After washing the membrane, add the secondary antibody, incubate at 37 °C for 1 h, wash twice, use the gel imaging system software to analyze the grey area of the protein band group in the film, and calculate the relative expression of AKT1 and MPK1 based on the internal reference GAPDH.

### Statistical methods

Statistical analysis was performed using SPSS 19.0 statistical software. The count data were expressed as mean ± standard deviation (x ± s). The comparison between multiple groups was performed by one-way analysis of variance, and *P* < 0.05 indicated that the difference was statistically significant.

## Results

### Collection of Cynarine targets and NAFLD-related genes

**Fig. 1:**
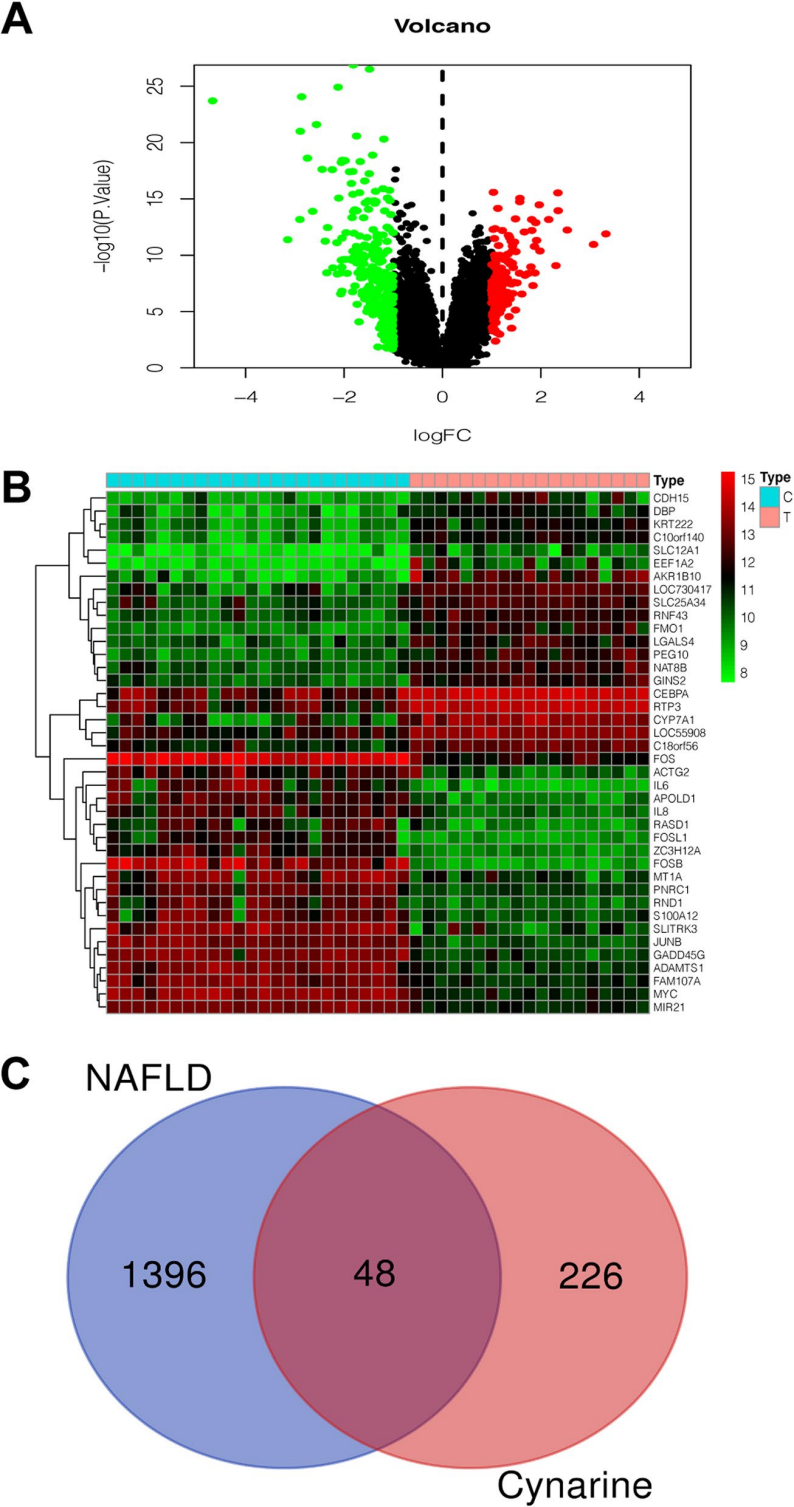
The 48 common targets for interaction between Cynarine and nonalcoholic fatty liver and disease. **A, B** Volcano plot of gene expression and heatmap of DEGs from GSE89632. **C** Venn diagram of Cynarine target and NAFLD-related genes

A total of 226 human proteins targeted by Cynarine were obtained from three public databases (Supplementary Material 1). 411 DEGs were obtained between patients with NAFLD and healthy donors, including 248 downregulated DEGs and 163 upregulated DEGs (Fig. 1A, B). Besides, another 1145 targets of NAFLD were screened from the Genecards, OMIM and CTD databases. Taking targets and DEGs together, there were 1396 potential NAFLD-related genes lastly (Supplementary Material 2). In the Venn diagram of targets genes (Fig. 1C), Cynarine targets and NAFLD-related genes shared 48 targets in common which might be the potential targets of Cynarine in treating NAFLD.

### PPI network construction

**Fig. 2:**
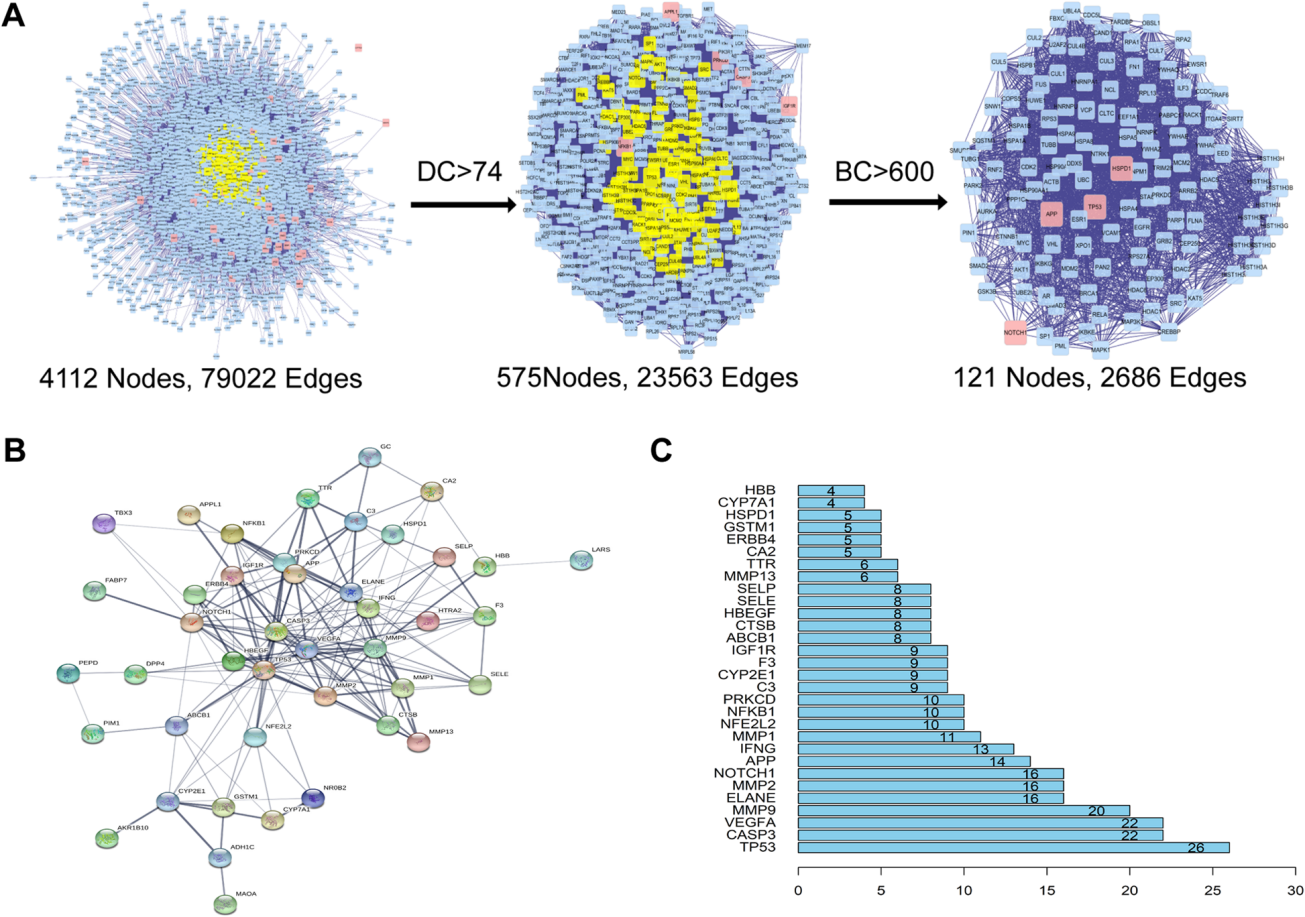
Protein-protein network (PPI) of candidate targets for Cynarine against NAFLD. **A** Topological screening of the interactive PPI network of Cynarine targets and NAFLD-related targets based on degree and betweenness centrality (BC). **B** PPI of potential therapeutic targets. **C** Bar plot of potential targets with degree value in PPI. Nodes represent targets; edges represent interactions between proteins

Firstly, we constructed PPI networks of Cynarine targets and NAFLD-related genes. Further, the two networks were merged, and a core network was obtained (121 nodes, 2686 edges) based on the DC and BC (Fig. 2A). Then we import 48 common targets into the string database with the organization as “Homo species” and medium confidence of Combine score between nodes as 0.400 (Fig. 2B). There were 48 nodes and 176 edges in the network. And the top seven proteins with high degree values were TP53, CASP3, VEGFA, MMP9, ELANE, MMP2, NOTCH1. which connected 26, 22, 22, 20, 16, 16, 16 proteins, respectively. These proteins were closely related to other targets and played a pivotal role in the entire regulatory network, which might be key targets of Cynarine in the treatment of NAFLD.

### Cynarine-targets-NAFLD network analysis

**Fig. 3:**
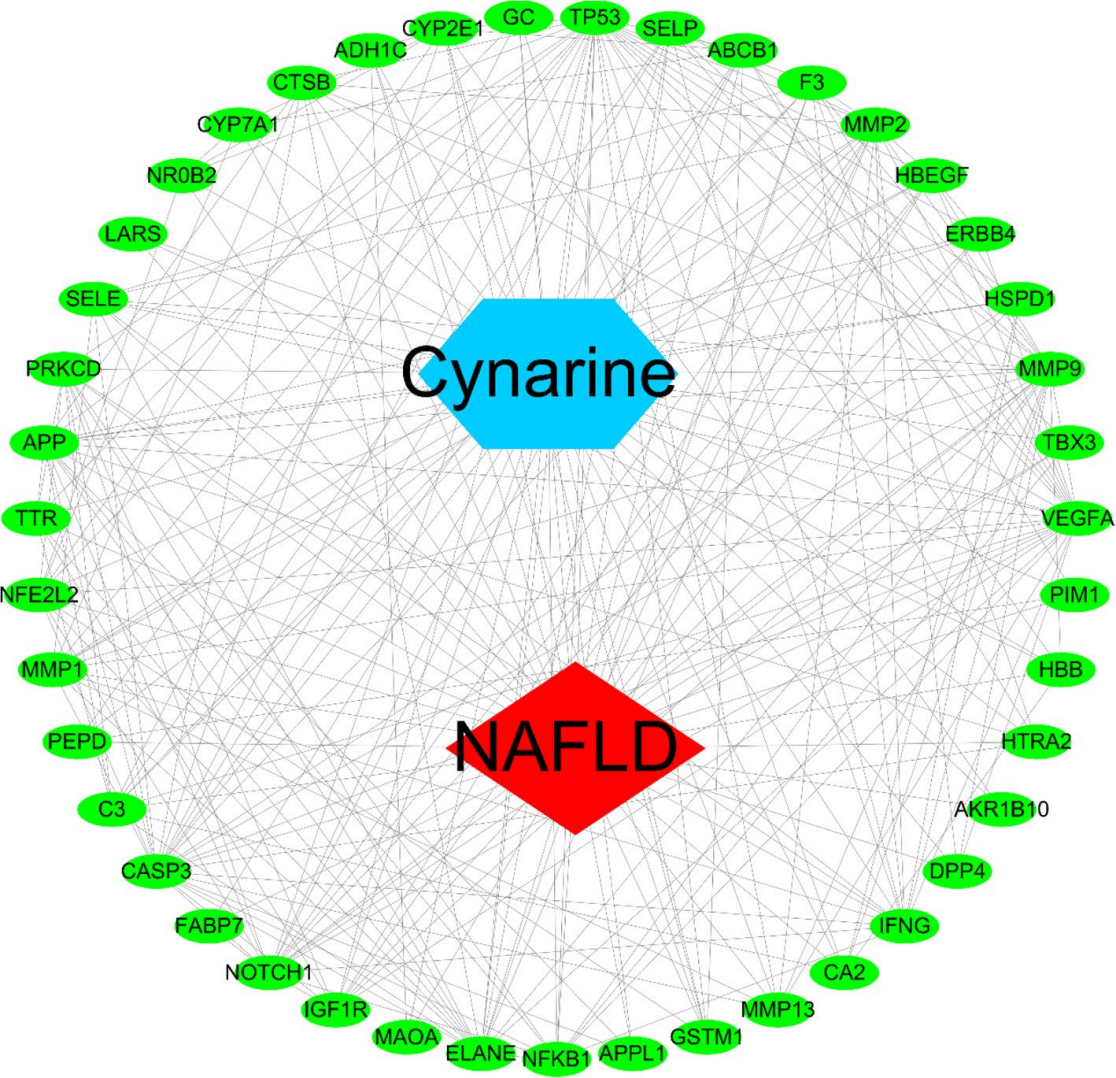
Cynarine-potential target genes-NAFLD network. The green node represents potential target genes; the blue represents Cynarine, and the red represents NAFLD

The information of Cynarine, potential targets, NAFLD were imported into Cytoscape 3.7.1 software to construct a network of “Cynarine-Targets-NAFLD”, which visually showed the relationship between Cynarine, 48 targets and NAFLD (Fig. 3).

### GO function and KEGG pathway enrichment analysis

**Fig. 4:**
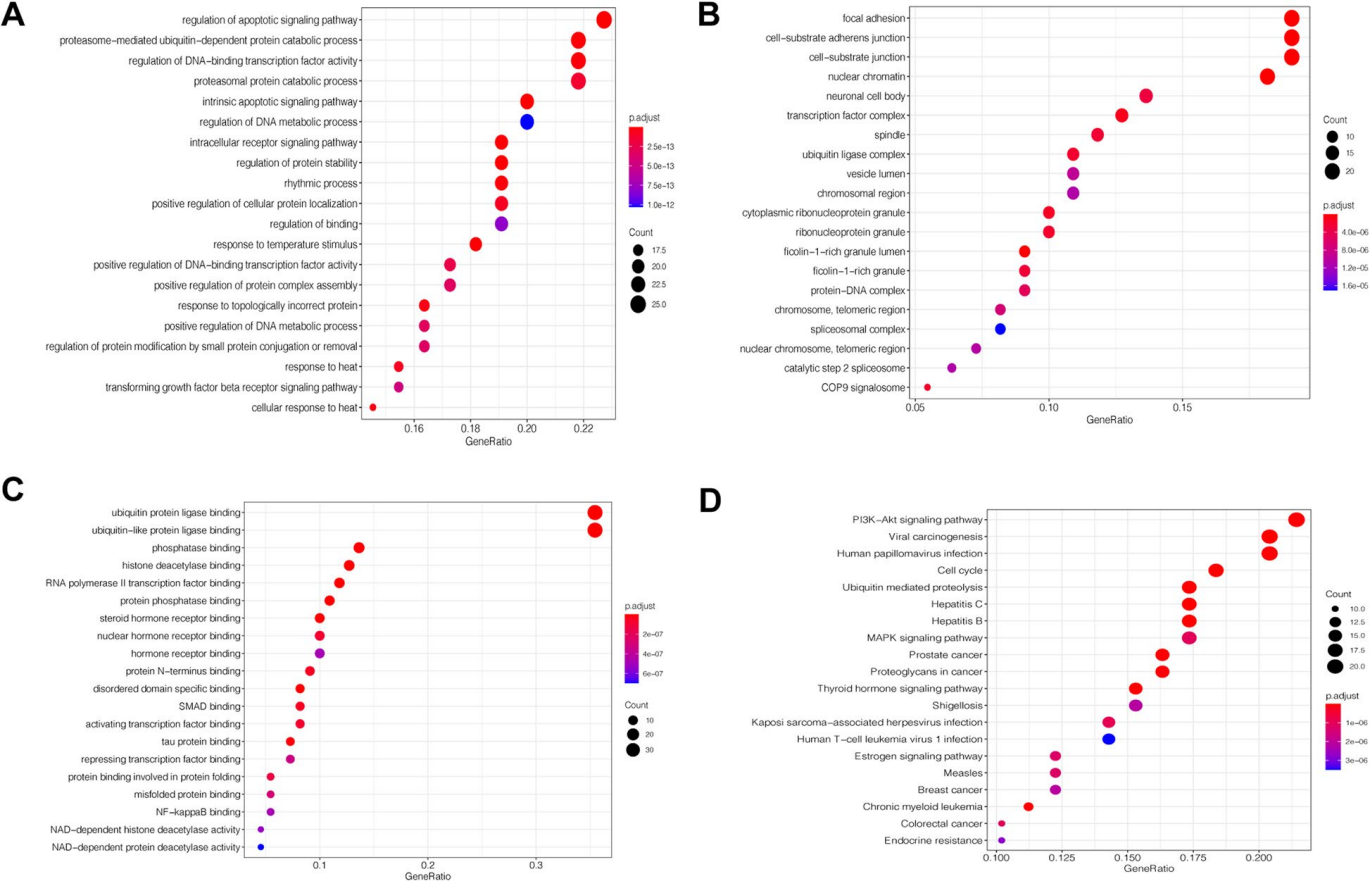
Gene Ontology (GO) and KEGG terms enriched by target genes. Dot plots of top 20 enriched biological processes (**A**), cellular component (**B**), and molecular function (**C**) terms with corresponding adjusted *p*-values analyzed by cluster Profiler. **D** dot plot of KEGG pathway terms enriched

To further analyze the gene function of the target proteins and the main signal pathways involved, we analyzed the GO function and KEGG pathway enrichment analysis of the 48 targets involved in the PPI network. The GO and KEGG enriched terms were measured by gene ratio, *P*-value and the number of genes in the regulatory pathway. Gene Ratio represents the degree of gene enrichment, and the *P*-value represents the significant difference of gene enrichment in each item. The enriched terms of biological process (BP), molecular function (MF), cell component (CC) and KEGG pathway were sorted according to *P*-value, and the top 20 terms were screened to draw bubble charts which were shown in Fig. 4A, B, C, and D. The top three enrichment terms in BP (Fig. 4A) were the regulation of apoptotic signaling pathway, intrinsic apoptotic signaling pathway and intracellular receptor signaling pathway. CC terms (Fig. 4B) mainly related to focal adhesion, cell-substrate junction and nuclear chromatin. The outstanding terms in MF (Fig. 4C) mainly involved ubiquitin protein ligase binding, histone deacetylase binding and disordered domain specific binding. KEGG analysis indicated that the targets of action involved 114 signal pathways. PI3K-Akt signaling pathway, cell cycle and ubiquitin mediated proteolysis were the top three enriched pathways that might serve as important pathways for Cynarine in the treatment of NAFLD (Fig. 4D). They were worthy of further discussion. Besides, the results showed that pathways related to immunity, inflammation, substance metabolism, and apoptosis also enriched more targets in pathway analysis, suggesting that regulation of immunity, anti-inflammatory effects, promotion of substance metabolism, and reduction of stem cell apoptosis might be one of the important pharmacodynamic mechanisms of Cynarine in the treatment of NAFLD.

### Target-pathway integration network analysis

**Fig. 5:**
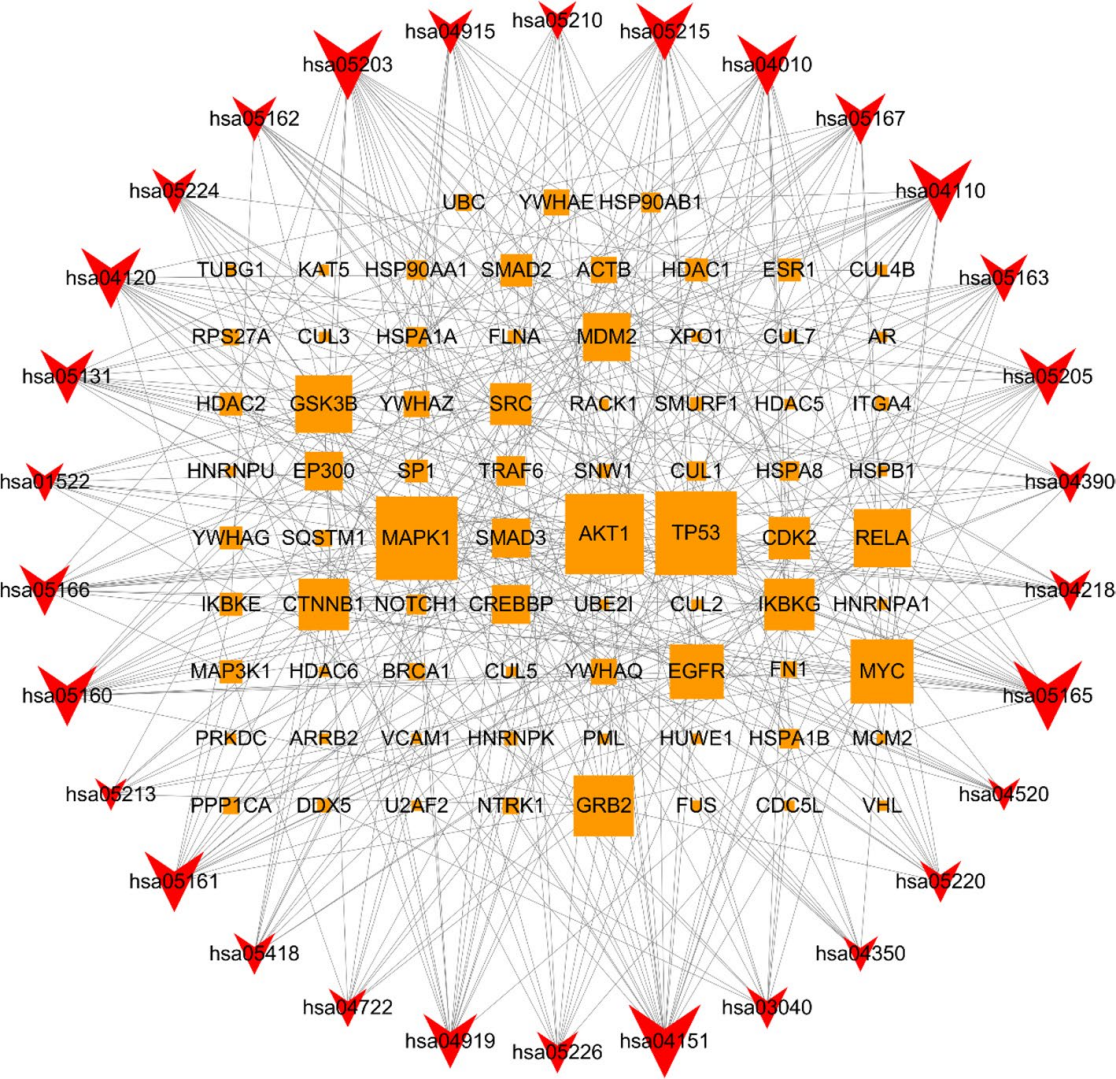
“Target-Pathway” network diagram

We imported the information of targets of action and their main signal pathways enriched into Cytoscape 3.7.1 software to integrate and construct a network of “Targets-Pathways”, which visually showed the relationship between 48 targets and 30 main signal pathways in Fig. 5. Further network analysis showed that the targets of MAPK1, TP53, AKT1 and MYC have the highest degree values, which are 24, 24, 23, and 18, respectively. Among the signaling pathways, PI3K-Akt signaling pathway, cell cycle and MAPK signaling pathway ranked at the top, which might be important pathways for Cynarine to play a role in treating NAFLD. Therefore, the multiple targets of Cynarine can participate in multiple signal pathways at the same time, forming an integrated network with multiple targets and multiple pathways.

### Molecular docking of Cynarine with core targets

**Fig. 6:**
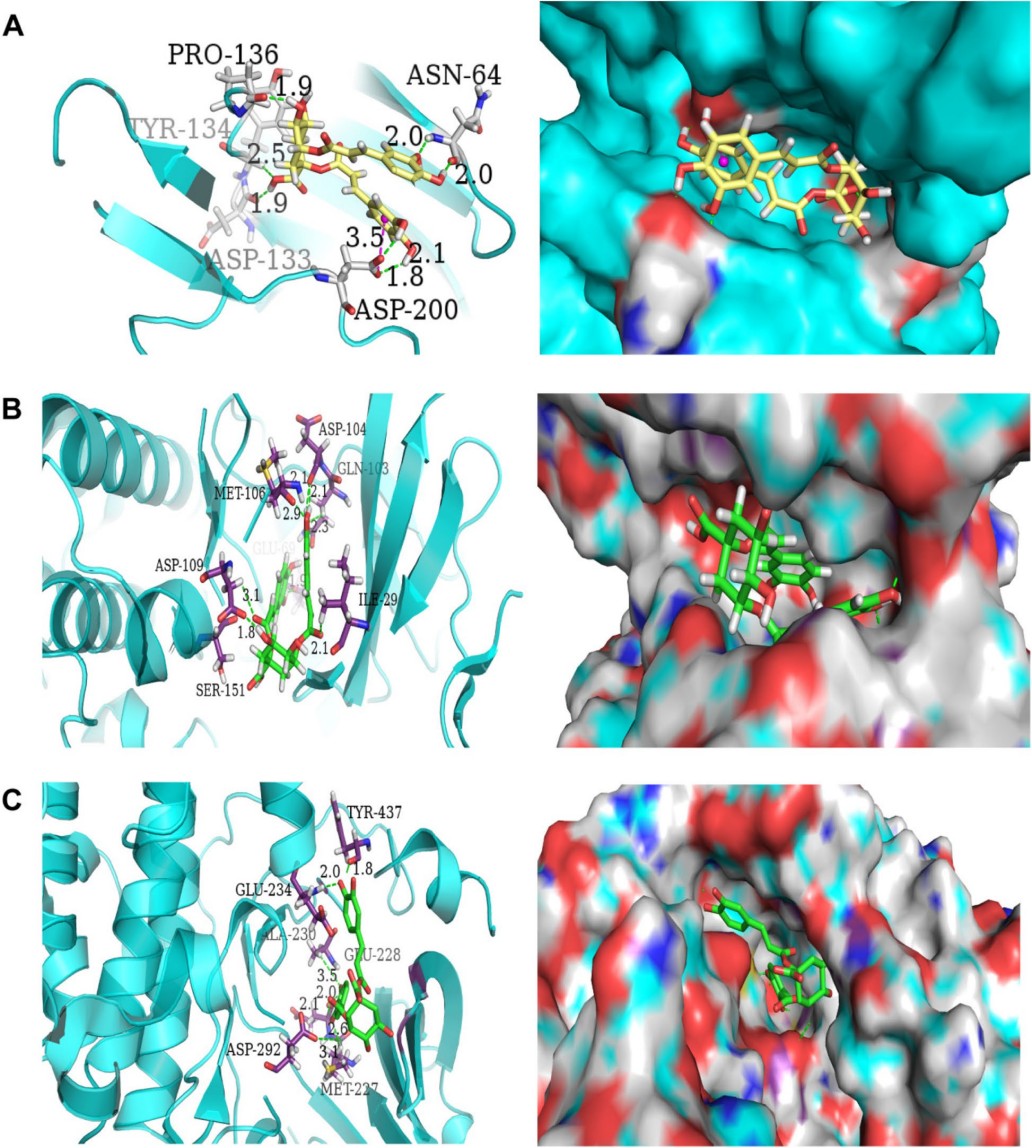
The docking conformation between Cynarine and major therapeutic genes. **A** GSK3B, **B** MAPK1, and **C** AKT1

To further verify the accuracy of the core targets (MAPK1, TP53, AKT1, MYC, GRB2, RELA, GSK3B, EGFR, IKBKG and CTNNB1) obtained in the network, the molecular docking analysis between them and Cynarine molecule were performed in sybyl 2.1.1. Eight of the ten core targets had docking scores of more than 5, demonstrating their good binding activity (Table 1). According to the docking score, the top 3 target proteins (GSK3B, MAPK1, and AKT1) were selected for visual analysis (Fig. 6). Figure 6 showed the non-bonding interactions, including hydrogen bonding (indicated by the green dashed line), π-π stacking effect (indicated by the purple dashed line) of the Cynarine molecule with the surrounding amino acid residues, and the space between the Cynarine molecule and the protein pocket position.

**Table 1:** The docking information of 10 targets with Cynarine

Targets	Docking score	PDBID
MAPK1	8.4546	4ZZN
TP53	4.8124	2FEJ
AKT1	7.0058	3OCB
MYC	5.0322	5YDE
GRB2	6.0258	3KFJ
RELA	7.504	3QXY
GSK3B	8.6478	4J1R
EGFR	6.957	5D41
IKBKG	4.6891	6MI3
CTNNB1	6.048	5IVN

### Cynarine inhibited fat deposition, reduced ALT and AST relief, and down-regulated AKT1 and MAPK1 expression in NAFLD model cells

**Fig. 7:**
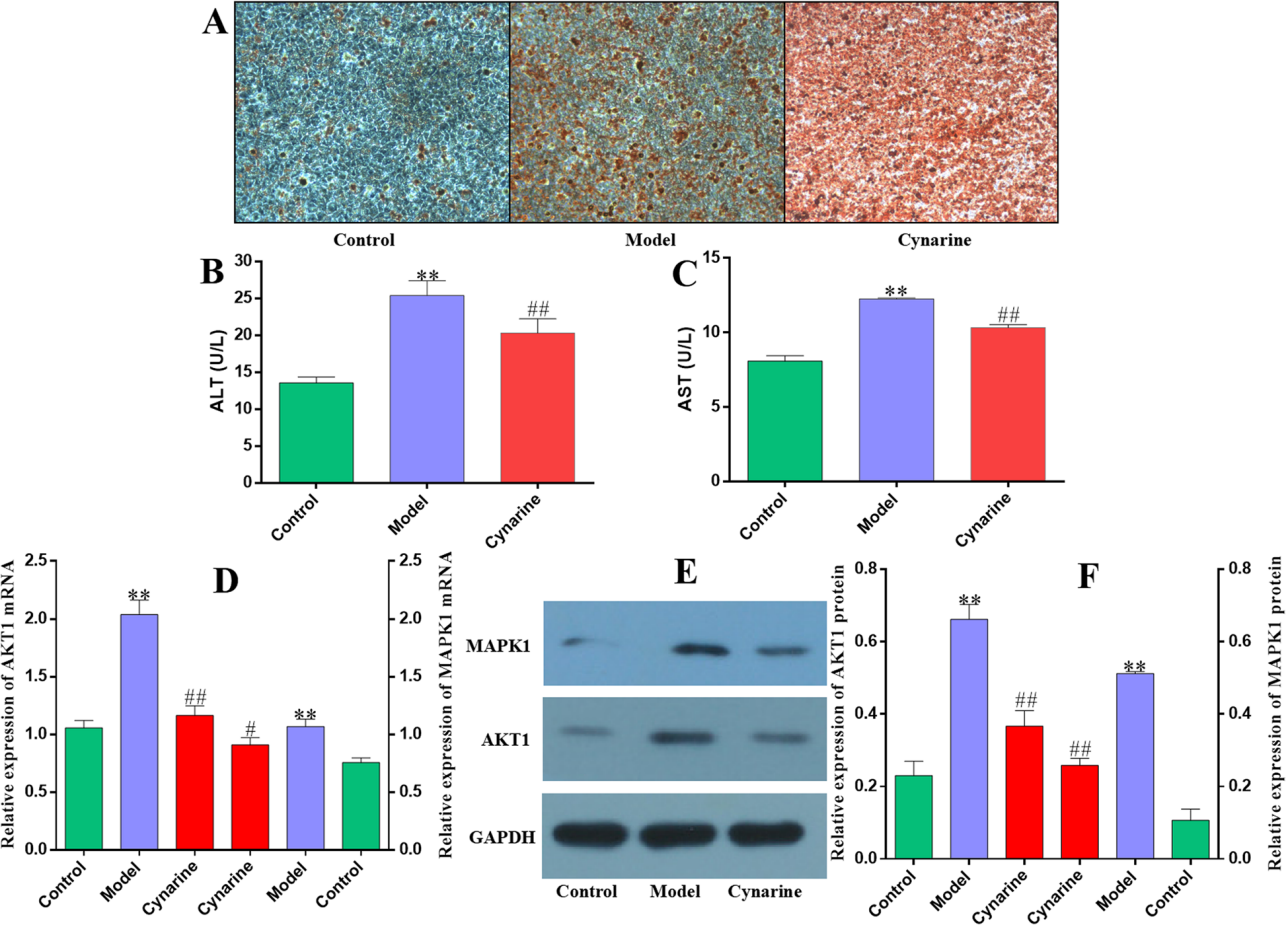
Cynarine inhibited fat deposition, reduced ALT and AST relief, and down-regulated AKT1 and MAPK1 expression in NAFLD model cells. **A** Comparison of oil red O staining and fat accumulation of cells in each group. **B, C** Comparison of the content of ALT and AST in the cell culture supernatant of each group. **D, E, F** The expression levels of AKT1 and MAPK1 in each group were detected. Compared with the control group: ^**^P<0.01; compared with the model group: ^#^*P*<0.05 or ^##^*P*<0.01

Figure 7A showed that the cells in the control group had clear edges, mostly polygonal or fusiform, arranged in a paving stone-like arrangement. The cells were tightly integrated, with small gaps, large nuclei, and very few red lipid droplets in the cells, while in the model group, there were few red lipid droplets in the cells. Red lipid droplets, some of the intracellular lipid droplets were located inside the cell membrane in a ring shape and squeeze the nucleus to one side. The cells were mostly round, and the cells were loosely combined. At the same time the accumulation of intracellular lipid droplets in the Cynarine intervention group was relieved. Figure 7B and C showed that, compared with the control group, the content of ALT and AST in the cell supernatant of the model group were significantly increased (*P*<0.01); compared with the model group, Cynarine reduced the content of ALT and AST (*P*<0. 01). Figure 7D, E and F showed the detection results of AKT1 and MAPK1 mRNA and protein expression levels. Compared with the control group, the expression of AKT1 and MAPK1 in the model group cells was significantly lower at the mRNA level (*P*<0.01); compared with the model group, AKT1 and MAPK1 expression levels were significantly reduced (*P* < 0.01) in Cynarine treatment group cells. Compared with the control group, the protein levels of AKT1 and MAPK1 in the model group were significantly reduced (*P* < 0.01); compared with the model group, the protein levels of AKT1 and MAPK1 in the Cynarine treatment group were significantly reduced (*P* < 0.01).

## Discussion

The advantages of TCM in the treatment of NAFLD have gradually emerged that single medicine or a combination of various medicines can regulate liver lipid metabolism, promote absorption and excretion, and avoid adverse reactions caused by long-term use of western medicine.

In this study, the targets of Cynarine were obtained by predicting and database searching. Then 48 targets for Cynarine in treatment of NAFLD were obtained by cross-mapping them with NAFLD -related targets in GeneCards, GEO, OMIM, and CTD databases. With the help of network pharmacology and network analysis, the “ Cynarine target- NAFLD target” network topology analysis was carried out. The PPI network was constructed, which showed that these target proteins did not act alone but had complex and interlocking interactions. The targets with high degree value had better connectivity which was essential for the entire regulatory network [18]. Therefore, they may play a central role in the treatment of NAFLD by Cynarine, which deserves our attention. The core targets include CASP3, TP53, VEGFA, MMP9, ELANE etc. CASP3 (caspase-3), which has the highest degree value, has been reported to a prominent role in hepatocyte apoptosis, fibrogenesis and fibrosis in a mouse model of nonalcoholic steatohepatitis [19]. It has been reported that p53 (TP53), another target p53 (TP53) with a high degree value, regulated various aspects of NAFLD development which was linked to insulin resistance, inflammation, lipid metabolism, and oxidative stress [20, 21]. Also, MMP9, ELANE, NOTCH1, with fewer connected targets, have all been proven to have important regulatory effects in reducing liver lipid accumulation, inflammatory responses, fibrosis, and improving insulin resistance [22, 23]. Pivotal targets in the PPI network were closely related to immunity and inflammation. Inflammation in liver cells led to the release of pro-inflammatory cytokines and the increase of apoptosis which was seen as a driving force for NAFLD progress [24]. The activation of immune cells could also trigger liver cell apoptosis, leading to tissue damage and laying the foundation for advanced fibrosis and liver cirrhosis [25]. Therefore, exploring inflammation and immune-related targets can improve the understanding of the underlying mechanisms of NAFLD and help the development of new therapies. The above research results indicate that the multiple core targets predicted in this study have an important role in regulating NAFLD, which is valuable for further research.

NAFLD is a complex disease, with lipid accumulation and inflammation being two important steps in its pathogenesis and progression, as well as two important links in the “second hit” theory. Therefore, reducing liver fat accumulation and reducing inflammation is the key to treat NAFLD.

The results of GO functional enrichment analysis in this study show that NAFLD’s therapeutic targets were mainly involved in the regulation of biological processes such as transcription, translation, and inflammation. These processes have the effects of improving metabolism, promoting tissue repair and reducing inflammation, and participating in the repair process of NAFLD diseased tissues from many aspects, which are consistent with the pathogenesis of NAFLD.

Analysis of target-pathway integration network based on the KEGG pathway enrichment showed that PI3K-Akt signaling pathway, cell cycle and MAPK signaling pathway were enriched by more targets, indicating that these three pathways played an important role in the treatment of NAFLD by Cynarine. The three pathways are closely related to the onset and progression of NAFLD. PI3K-Akt signaling pathway, which is a significant pathway for the metabolism of glucose and lipids, and understanding this pathway’s role in NAFLD is crucial. It has associated targets with MAPK pathway, including the predicted key targets such as GSK3, MYC and IKBKG, which can be abnormally activated by various toxic insults or cellular stimuli related to NAFLD [26–28]. Cell cycle was closely related to the metabolic regulation of NAFLD [29]. A study demonstrated that the inhibition of MAPK signaling pathway decreased hepatocyte apoptosis and improved hepatic fibrosis in rats with NAFLD [30]. In addition, KEGG pathway enrichment analysis revealed that proteoglycans, the thyroid hormone signaling system, and human cytomegalovirus infection are all intimately associated to NAFLD [31, 32]. They were all enriched with more Cynarine therapeutic targets, suggesting Cynarine may interfere with inflammation and immunity, metabolic homeostasis, lipotoxicity, liver cell death and other pathogenesis and progression mechanisms in NAFLD by regulating these pathways.

Cell experiment results showed that Cynarine could reduce the fat deposition ability of NAFLD model cells, and effectively reduce the levels of ALT and AST released by liver cells due to excessive lipid accumulation. Additionally, we discovered that Cynarine can also inhibit the expression of AKT1 and MAPK1 [28, 33, 34].

## Conclusion

In summary, this study used network pharmacology to construct a network of Cynarine-targets of action, which included 48 potential targets. Analysis showed that CASP3, TP53, MMP9, ELANE, NOTCH1 played a prominent role in many targets. It may be the key to Cynarine’s prevention and treatment of NAFLD that PI3K-Akt signaling pathway, cell cycle and MAPK signaling pathway and other potential signal pathways were regulated to interfere with inflammation and immunity, metabolic homeostasis, lipotoxicity, liver cell death and other mechanisms. Also, this study revealed that Cynarine could significantly reduce the fat deposition ability of NAFLD model cells, which may be closely related to the effective regulation of AKT1 and MAPK1 expression by Cynarine. In the later stage, we will start from animal level and clinical trials to further verify the effectiveness of Cynarine in the treatment of NAFLD and other potential mechanisms, and provide more reliable ideas for the treatment of NAFLD.

## Supplementary Information

**Additional file 1.**

**Additional file 2.**

**Additional file 3.**

## Data Availability

The total of 226 human proteins targeted by Cynarine are presented in Supplementary Material 1; The 1396 potential NAFLD-related genes are presented in Supplementary Material 2.

## Abbreviations

NAFLD: Non-Alcoholic Fatty Liver Disease
GEO: The GEO database (https://www.ncbi.nlm.nih.gov/geo/)
DEGs: Differentially Expressed Genes
OMIM: The OMIM database (https://omim.org/)
CTD: The CTD database (http://ctdbase.org/)
PPI: Protein-protein interaction
GO: Gene Ontology
KEGG: Kyoto Encyclopedia of Genes and Genomes
ALT: Alanine transaminase
AST: Aspartate transaminase
PVDF: Polyvinylidene fluoride
BP: Biological process
MF: Molecular function
CC: Cell component
