# Supplementary material for: Mechanisms of Cynarine for treatment of non-alcoholic fatty liver disease based on the integration of network pharmacology, molecular docking and cell experiment
Source: Hereditas. 2022 Dec 1;159:44. doi: 10.1186/s41065-022-00256-7 (PMC9714250; doi:10.1186/s41065-022-00256-7)
Supplement: Supplementary file 1 — Additional file 1. [file 41065_2022_256_MOESM1_ESM.pdf]

PCNA
CSDE1
INADL
RUVBL1
GPD1L
FES
PRKD2
INHBA
RNASE2
TRIP4
SPEN
EXOSC9
SEC13
ZBTB43
ZEB2
CPSF3
EPN1
UAP1
PSMD4
MEF2A
PMVK
SRM
UEVLD
KYNU
CTSB
NCAM1
DOK2
DCC
MAP3K3
CDC14B
GPX3
RPGRIP1L
KIF11
PTPRF
EFNA1
GZMK
LRBA
ITPKC
ARHGAP5
RND1
DAZAP1
MMP2
TRIM21
PECR
CD3E
RAD23B

FLNB
AOC3
DHRS1
NMRK1
HBEGF
HECTD1
GZMB
EPHA3
ZNF462
RPA3
HCK
TRIM29
PRPSAP2
RAN
PANK1
ASAP1
PDK2
FCGR2A
DPP4
SEC23A
CDK5R1
MEMO1
POU2F1
TASP1
MDH2
TP53
PCCA
TIMM9
HDAC7
HIBCH
MEPCE
ST18
FABP7
SERPINB3
REPS2
ACAT2
HDX
EZR
CYP2E1
ITPK1
PTPN14
ANXA3
PC
RAB26
NCBP1
TBX3

TRIO
GALK1
GCH1
AMY1B
NELFE
PDK3
IRAK4
INMT
RUNX1T1
CYP2A13
PAPSS2
FH
KDR
HSCB
NT5C3A
MMP3
ACVR2B
SKP1
NME3
NCOA3
NPR3
USP5
PPP1CB
PYGL
NFATC1
ACADVL
GSTM1
IGF1R
RPS6KA5
SMG7
SNX22
NUP153
IDE
MAGEA4
MMP1
TPP1
ZCWPW1
ANXA6
TLR1
FABP3
SMURF2
APPL1
BMP1
C3
HAT1
HBB

MSN
TXNDC12
TIMM44
GP6
CRABP2
LARS
KLF10
MATK
IMPA1
SH3BGRL2
SHFM1
IFNGR1
NOTCH1
GSN
BLVRB
MAOA
TOP1
CYP7A1
XRCC6
SF3A3
VEGFA
TNPO1
PAH
CUX2
DAPP1
GCDH
CDK2
MSH2
F3
HPD
PEPD
AASDHPPT
CA8
ZHX3
CSF2RB
HLA-G
SUPT5H
LSS
RARA
CYP2R1
IMPDH1
HSH2D
PARN
ACACB
CSNK1G2
MERTK

AKR7A3
GC
YY1
NSUN5
TRAF3
MAGI2
SMAP1
IGHG1
RRM1
MDH1
EMILIN1
PTK2B
ITGB3
MYSM1
B2M
PDZD3
NAA50
HK2
HTRA2
ERBB4
RTN4IP1
OXSR1
DLAT
AK5
GSKIP
DHX30
CRYM
GSTT2B
ASMTL
GALNT10
CKM
DGKA
S100A6
COBLL1
WARS
BCHE
ADH1C
TAF13
ODC1
HYAL1
GAD2
GART
CAMK4
TAB1
IFNG
VPS26A

IMP3
PGC
CSNK1G1
B3GAT2
NR0B2
SLC37A4
AKR1B10
AKR1C4
CA6
GLO1
CA5B
NFKB1
CA5A
MAPT
APP
CA7
TTR
HSPD1
NFE2L2
CA14
MMP9
AKR1B1
APP
AKR1B1
AKR1B10
MMP12
MMP2
MMP13
SLC37A4
PYGL
ELANE
ABCB1
FYN
TTR
MMP1
PRKCD
CA2
CA7
CA12
CA4
CA1
HCAR2
CA6
CA13
CA5B
CA5A

FOLH1
NEU4
SELE
SELP
PDE4D
PDE9A
PDE1B
PIM1
CASP3
CD22
