# Supplementary material for: Mechanisms of Cynarine for treatment of non-alcoholic fatty liver disease based on the integration of network pharmacology, molecular docking and cell experiment
Source: Hereditas. 2022 Dec 1;159:44. doi: 10.1186/s41065-022-00256-7 (PMC9714250; doi:10.1186/s41065-022-00256-7)
Supplement: Supplementary file 2 — Additional file 2. [file 41065_2022_256_MOESM2_ESM.pdf]

AXUD1
GADD45B
MIR21
JUNB
FOSB
MYC
FOS
PPP1R15A
JUN
CEBPD
GADD45G
PIM1
FOSL2
AGPAT9
SOCS2
ADAMTS1
FAM107A
CYR61
EPHA2
PPRC1
MAP3K8
SOCS3
ARID5B
NFIL3
KLF6
RFXDC2
CCL2
FMO1
HBEGF
PELI1
PNRC1
WNT5A
ELL
BCL3
KLF11
RGS1
TYMS
IL4R
FILIP1L
GINS2
JUB
BCL6
PHLDA1
C10orf140
RGS2
SERTAD1

APOLD1
THBS1
ITPRIP
IER3
C13orf15
TMEM169
SLITRK3
PEG10
C18orf56
NAT8B
FOXO1
ZC3H12A
BCAR3
ETS2
HMMR
P4HA1
LOC730417
RNF43
FLJ42280
LOC55908
PTGS2
SPRY4
C10orf10
AKR1B10
C21orf63
NFKBIZ
ASPM
EGR1
SIK1
SNORD11B
TBX10
S100P
FBXO27
IL6
SNORA41
KRT222
GRIA3
FOSL1
PFKFB3
C8orf47
IL8
HIVEP1
CRISPLD2
CYP7A1
CISH
NOL3

RALGDS
CKAP2
KCNK1
LGALS4
C17orf96
SLC2A3
SDC4
EMP1
PIM3
ZFP36
PLAUR
TNFRSF1A
RTP3
NR4A1
C1orf203
C11orf52
SIPA1L2
IRAK3
IFIT2
THBD
KLF5
PADI4
MAL2
ISM1
C9orf125
C19orf25
FLJ36031
GPRC5A
RRS1
RAB26
IFIT3
KIAA0040
RMND1
CLCF1
PROK2
FBXO2
IL1RL1
MAFF
SOCS1
ODZ3
IGFBP1
HAS2
FRAT1
NR4A2
ME1
C5AR1

NFE2
IL1B
PRSS3
FRMD7
FPR1
MOGAT2
C14orf143
C9orf91
EEF1A2
CDC42EP3
BACH2
TLR7
SLC25A34
SNORA1
TMEM20
ARL14
RTP4
RND1
ANKRD29
NLF2
MIDN
EPHA1
SERPINE1
TNFSF14
FLJ41603
IL1RN
SLC7A6OS
VNN3
DIO3OS
MT1A
ACTG2
DBP
PTTG1
C14orf80
MIR146B
CEBPA
C6orf129
IGFBP2
EDAR
S100A12
PAQR7
PTGER2
KLF4
SLC12A1
GJA1
KIAA0133

C2CD4B
TUBB6
RALGPS1
ELF3
MIR564
NCOA7
FOXC1
ZNF878
SIRT4
FAM168A
DGCR5
HDC
SLC11A1
SPSB1
C19orf44
GPAM
LOC730101
SNORD11
PPAN
ANXA13
IL8RB
MMP19
RRP12
TBX3
TRIM15
SNORA67
TNFSF10
IL18RAP
MGC13057
SPATA18
ANKS4B
C2orf54
URB2
ONECUT1
NINL
SPTLC3
IL10
HIST1H2BF
NAGS
CDH15
TGFB3
TMEM63A
IER5L
NR4A3
PRO0628
ELFN1

LONRF2
CHRNB1
STC1
TAGLN
CNTNAP2
FCGBP
PAQR4
SERPINB9
LOC148413
GRAMD4
ZNF219
AMOT
CMTM2
TNFRSF10D
DLEU1
MGC12965
GPER
ODF3L1
LIF
C19orf23
MYBPH
C12orf27
PITPNM2
MTMR4
TNFRSF12A
LOC154761
CD3G
GFPT2
FABP4
DKFZP564J102
CENPQ
TMEM233
PCDH24
RASD1
CRYAA
FADS1
LOC201229
ABCC6P1
PNPLA3
ACE2
AVPR1A
MAP3K14
KIAA1161
AFP
ANXA9
FGF14

LRRC31
XKR4
FLJ45139
GIMAP2
TMEM92
BATF3
MIR886
ZNF295
HIST1H2AC
TMEM45B
ITLN2
TMEM98
MAMDC4
DKFZp686O24166
TRHDE
TLN2
PIGR
MLF1
FREM2
CXCL9
S100A8
ORAI3
HTR2B
SLC22A11
LOC388796
TNFAIP8L3
ADAMTS4
C1orf51
BIRC3
ABCG8
SLC16A13
GPR3
MCAM
CNN1
TNFAIP6
SOX17
FAM124B
SPHK2
DHODH
LOC100131726
PTX3
TCAP
ANKRD56
SLC25A42
JAKMIP2
FRMD6

UBD
RBP5
ALDH1A3
MYH11
TMPRSS2
NCAPG2
TIPARP
CD274
KCNE4
NOL4
LRIG3
FADS2
OSM
SNORA71A
SBNO2
APOBEC3A
SLC7A1
FJX1
PROL1
KIF22
TBX15
OXCT2
CCL3
USP18
SNORD53
FAM169B
C20orf77
EFHD1
RAX2
ARID3C
FAM47E
C2orf82
SNORA75
VCAN
EPB49
ABHD8
RGS16
PAPPA
CHST9
IRF1
IRS2
ROM1
C19orf59
ACSM1
C7orf54
CIT

MT1B
COL5A3
C2CD4A
SERPINB2
DHRS13
FLNC
BCL2A1
DAND5
C15orf52
KANK4
VIL1
C6orf142
DCDC2
CCL20
MAPK4
SHD
CH25H
JMJD5
LRRC19
GFRA3
MIR221
SNORA11
SNORA36B
MIOX
SAA1
PJCG6
LIN28
DEFA1
DEFB118
LOC650293
SBK2
OR4F5
POM121L4P
GAGE2B
MIR548I1
HLA-C
SNORA17
GAGE12C
GAGE12J
OR4F21
GAGE4
GAGE5
REXO1L1
SREBF1
PPARA
CPT1A

TGFB1
NFE2L2
CAT
ADIPOQ
LEP
SCARB1
LDLR
CYP2E1
PDK4
NQO1
NR1H4
ABCC2
VLDLR
AHR
CD14
FAS
TRIB3
PRKCA
CYP1A2
XBP1
ALDH1A1
GSTP1
IL1A
GSTA1
IL4
ALDH2
PNPLA3
PTEN
SERPINB2
CSF2
PEMT
PRKACA
STC2
NR5A2
GSTM1
ACE
CYP17A1
ABCB4
GSTT1
AHCY
ALDH4A1
LIF
PRKCD
GNMT
MMP1
JAK2

F2
TNFRSF1B
IKBKG
PRKCE
ALDH1B1
FOLR2
KLB
TM6SF2
ADH4
ATP5B
LAMA1
B3GAT1
EIF2AK1
PRF1
RDX
ADH1B
IL3
ADH1A
RAG2
PPARD
FGF21
SIRT1
AD7CNTP
IBD7
PLEKHG5
KIAA0720
DSMA4
CMTRIC
DJ1
PARK7
KIF1B
CMT2A
CMT2A1
NBLST1
MTHFR
MFN2
KIAA0214
CMT2A2A
HMSN6A
CMT2A2B
GBD2
PINK1
PARK6
ECE1
ALPL
HOPS

TNSALP
RHCE
RHNA
FABP3
YARS1
YARS
CMTDIC
TYRRS
YTS
YRS
GBD3
ELOVL1
SSC1
IKSHD
FAAH
FAAHP1
FAAHOUT
LINC00505
PARK10
AAOPD
PAOD1
FOXD3
AIS1
VAMAS2
DNAJC6
DJC6
KIAA0473
PARK19
IL23R
IBD17
TNNI3K
CCDD
ABCA4
ABCR
STGD1
FFM
RP19
CORD3
ARMD2
AGL
GDE
DBT
BCATE2
ATP1A1
CMT2DD
HOMGSMR2

AD13
NBLST6
NOTCH2NLC
NIID
ETM6
ECM1
URBWD
ZNF687
KIAA1441
PDB6
SLC27A3
FATP3
MUC1
PUM
MCKD1
GBA
PKLR
PK1
LMNA
LMN1
EMD2
FPLD2
CMD1A
HGPS
COPA
AILJK
MPZ
CMT1B
CMTDID
DSS
CHN2
FMO1
FMO4
AD14
NCF2
CELIAC7
IBD23
PARK16
INAVA
C1orf106
IBD29
IL10
CSIF
GVHDS
RMD1
TLR5

TIL3
SLEB1
MELIOS
PSEN2
AD4
STM2
CMD1V
ARV1
EIEE38
ADAM17
TACE
NISBD1
NBAS
NAG
SOPH
ILFS2
APOB
FLDB
LDLCQ4
FCHL2
HADHA
MTPA
MPV17
MTDPS6
CMT2EE
ABCG8
GBD4
STSL1
PARK3
HTRA2
OMI
PARK13
PRSS25
MGCA8
FABP1
CELIAC8
COPD
ZAP70
SRK
ADMIO2
IMD48
LCO
CHDS2
HOXD10
HOX4D
PDE11A

PDE11A1
PDE11A2
PDE11A3
PPNAD2
BMPR2
PPH1
POVD1
CTLA4
IDDM12
CELIAC3
ALPS5
FARSLB
FRSB
RILDBC
IRS1
SP110
IFI41
IFI75
VODI
GIGYF2
KIAA0642
PARK11
ATG16L1
APG16L
IBD10
SAG
RP47
AGXT
SPAT
PDCD1
SLEB2
IBD9
MYMY1
MYMY
CAV3
LQT9
MPDT
RMD2
CX3CR1
GPR13
V28
CELIAC9
HSCR6
IBD12
GBE1
GSD4

APBD
CHDS5
POGLUT1
CLP46
KTELC1
RUMI
C3orf9
DDD4
LGMDR21
RAB7
CMT2B
PSN
AD15
PARK21
ATP2C1
BCPM
HHD
DZIP1L
DZIP2
PKD5
GYG1
GSD15
CELIAC10
MME
CD10
CALLA
NEP
CMT2T
SCA43
GNB4
CMTD1F
EIF4G1
EIF4G
PARK18
DNAJB11
HEDJ
DJ9
ABBP2
PKD6
CELIAC11
HTT
HD
IT15
LOMARS
HDL3
HLN2

PROM1
PROML1
AC133
RP41
CORD12
CD133
MCDR2
STGD4
UCHL1
PARK5
SPG79
NDGOA
PHOX2B
NBPHOX
PMX2B
NBLST2
CCHS
AIS4
VAMAS5
PKD2
PPM1K
PP2CM
PTMP
MSUDMV
SNCA
NACP
PARK1
PARK4
ADH1C
ADH3
ELOVL6
LCE
FACE
FABP2
CELIAC6
AIS5
FAT4
VMLDS2
HKLLS2
HSCR9
TRIM2
KIAA0517
CMT2R
FAT1
FAT
GDNF

HSCR3
IBD18
ELOVL7
HEXB
PDE8B
PPNAD3
ADSD
APC
GS
FPC
BTPS2
DESMD
AITD2
CELIAC2
IBD5
PDB4
LEAP2
SAR1B
CMRD
SARA2
ANDD
HARS1
HARS
USH3B
CMT2W
LARS1
LARS
LFIS
ILFS1
SH3TC2
KIAA1985
MNMN
IRGM
LRG47
IFI1
IBD19
FAT2
MEGF1
SCA45
FABP6
ILLBP
SQSTM1
P62
PDB3
FTDALS3
NADGP

DMRV
HULC
ELOVL2
SSC2
HFE
HLA-H
HFE1
MVCD7
TFQTL2
IBD3
VAMAS6
DHX16
DDX16
DBP2
PRP8
NMOAS
HLA-B
SPDA1
HLA-DQA1
CELIAC1
HLA-DQB1
HLA-DPB1
AD17
FCYT
PKHD1
ARPKD
PKD4
ELOVL5
HELO1
SCA38
AITD1
SLC17A5
SIASD
SLD
COX7A2
ELOVL4
ADMD
STGD2
STGD3
ISQMR
SCA34
BCKDHB
E1B
SEC63
PCLD2
FIG4

KIAA0274
SAC3
ALS11
YVS
BTOP
FABP7
FABPB
BLBP
ARG1
ENPP1
PDNP1
NPPS
M6S1
PCA1
ARHR2
COLED
CELIAC12
LPA
PRKN
PARK2
PDJ
TBP
SCA17
HDL4
IL6
IFNB2
BSF2
HSF
HGF
HNRPA2B1
IBMPFD2
GARS1
GARS
SMAD1
CMT2D
HMN5
SFRP4
FRPHE
PYL
PGAM2
PGAMM
GSD10
EGFR
NISBD2
CHCHD2
PARK22

NCF1
CGD1
HSPB1
HSP27
CMT2F
HMN2B
CD36
CHDS7
BDPLT10
ABCB4
PGY3
MDR3
ICP3
ABCB1
PGY1
MDR1
IBD13
CLCs
PON1
PON
ESA
MVCD5
PON2
IBD11
ARPC1B
ARC41
PLTEID
RINT1
ILFS3
IRF5
IBD14
SLEB10
AD10
NOS3
PRKAG2
WPWS
CMH6
AIS2
VAMAS3
GATA4
ASD2
VSD1
TACHD
TOF
CHDS9
NEFL

CMT2E
CMT1F
CMTDIG
AD12
CMT2H
JPH1
JP1
CMT2K
GDAP1
CMT4A
CMTRIA
FABP5
PAFABP
EFABP
PMP2
CMT1G
FABP4
FABP12
CDH17
CDH16
HPT1
MYMY3
TNFRSF11B
OPG
OCIF
PDB5
TNF
INS
IL6
PNPLA3
PPARG
GGT1
LEP
SLC17A5
IL1B
TLR4
IL10
FAS
APOB
AKT1
NR1H4
HFE
ALB
APOA1
GPT
RETN

HADHA
CAT
ADIPOQ
MTTP
APOE
INSR
IRS1
RBP4
LPL
LEPR
MIR122
NR1H3
MLXIPL
PPARA
CRP
SERPINE1
PPARGC1A
DDIT3
TGFB1
TP53
AHSG
MIR21
PTEN
CXCL8
IRS2
F2
CYP2E1
APOC3
PON1
MIR34A
FASN
UCP2
IFNG
MIR17
SREBF1
KRT18
IGF1
FABP1
CASP3
CPT2
VEGFA
MIR140
MIR126
IL1A
MIR132
CCL2

AFP
ACE
MTHFR
MAPK8
SIRT1
MIR30A
SLC2A4
MIR22
CTLA4
APP
MIR27A
STAT3
HNF4A
SOD1
MIR31
TLR2
MIR10B
TM6SF2
ABCA1
SERPINA1
ICAM1
MIR200A
EGFR
SLC25A13
MIR200B
TIMP1
MIRLET7D
CTNNB1
FASLG
MFN2
LMNA
MIR26A1
ATP7B
CPT1A
MTOR
MIR200C
HMGCR
MIR130A
MIR183
RELA
MIR107
MIR29C
MIR33A
TNFRSF1A
MIR16-1
MIRLET7E

CAV1
MIR203A
ADIPOR1
MSR1
MMP2
TF
ABCB11
PIK3CA
LDLR
SMPD1
NAFLD1
XBP1
JUN
ACACA
HGF
CP
SOD2
HMOX1
CDKN2A
ADIPOR2
HLA-DQB1
FGF21
CD36
VWF
IL1RN
CFTR
MIRLET7C
MIR451A
CETP
MYC
TTR
SREBF2
MPO
NOS2
ABCB4
LCAT
MIR125A
TERT
EGF
LIPA
ESR1
MIR30D
MIR378A
MMP9
NOTCH1
VDR

NDUFS1
NFKB1
MAP3K5
CYP3A4
CCND1
CASP8
MARS1
SQSTM1
SPP1
G6PC
ABCC2
GCKR
GCG
DPP4
BAX
MIR483
NAFLD2
BDNF
SMAD4
CDKN1A
BCL2
STAT1
VIM
SERPINC1
NOD2
PTGS2
MIR429
ELANE
NAMPT
BMP6
LIPC
MMP1
VCP
THBD
HAMP
PTPN11
CXCR4
RARRES2
BSCL2
JAG1
PNPLA2
PCSK9
PIK3R1
TNFRSF1B
GANAB
POMC

HSPB1
MT-TK
AKT2
IGF1R
UGT1A1
CCN2
C3
MIR155
NGF
GH1
HP
NR1H2
NDUFS4
FN1
SLC6A3
FGF19
DRD2
GHRL
NLRP3
IL18
FABP2
SERPINA3
TNFSF11
TNFRSF11B
ENPP1
CFH
IFNA1
SLC2A1
CYP1A2
ABCB7
MIR181B1
PRKAG2
MIR146A
LIPE
MDM2
HSPD1
TGFBR2
FLT1
APOA5
CDK4
NCAN
PKLR
XDH
SDHB
MT-CO1
CTSD

CYP1A1
IL17A
KRT8
PRKAB1
CCL3
CYP7A1
DNMT1
GSTM1
F3
PC
PRMT7
SOCS3
FABP4
SLC6A4
PLAU
GCK
GSTP1
ASS1
PDGFRB
SMAD3
PRKAA1
CDKN3
TARDBP
CXCL10
SDHD
BGLAP
DNAJB11
NDUFS3
ALPP
CCL5
AGPAT2
GRN
FGF23
AGTR1
SCD
SDHC
COX5A
ALDH2
GSK3B
EZH2
VCAM1
NDUFS2
FOS
SHBG
FABP3
MT-CYB

HBB
MYD88
HIF1A
CASR
CYCS
SDHA
SELE
LOX
APOA2
EP300
HTR2A
TGFBR1
CYBB
IDH1
SPARC
SMARCA4
LCN2
MT-CO2
ATF4
CHI3L1
AGT
NDUFS7
CNR1
SELP
PLIN1
MIR151A
CTSB
RAC1
CD44
MIR29A
NFE2L2
SLC40A1
MT-ND6
EIF2AK3
GLUD1
HSD11B1
MAPK14
MIR20A
NPPB
CRAT
KRT7
MIR221
NR1I2
MIR192
KRT19
AKT3

SCARB1
MEG3
NDUFV1
TFR2
MIR181A1
RXRA
ADH1B
TIMP2
TNFSF10
CPOX
FADS2
LPA
SMAD7
MIR214
PRL
ADH1C
MIR144
TNFAIP3
ALPL
CYP2A6
TLR9
CYP17A1
NDUFV2
IGFBP3
ACTB
NDUFA13
NDUFB8
FABP12
MIR195
PARP1
ERCC2
CHIT1
NDUFA9
MT-CO3
CRH
CYP1B1
PLA2G4A
IL6R
NDUFB9
NDUFA6
MIR27B
DSP
EPAS1
IFNL3
CA2
MIR222

IRF1
SPINK1
PIK3R2
CES1
EIF2S1
NPY
CYBA
MIR182
PPIG
S100A9
MMP13
MIR15B
CD55
FGFR4
UCP1
SLC27A1
CHUK
CLU
NDUFS8
ERBB4
FGA
IGFBP1
HJV
BIRC5
OGG1
ETS1
GSTT1
CD14
SUMF1
BMPR1A
IL1R1
MAPK10
SI
NDUFA1
COX6B1
CDC42
GC
LRP6
MYH9
NDUFS6
SERPINA6
PRKAA2
HSPA5
DRD3
NDUFA2
LNCARSR

NDUFB11
LEPQTL1
GHR
MIR154
AKR1A1
CEBPA
CXCR3
ADRB2
CCK
CASP9
HNRNPA1
CSF1
MT-ND3
IKBKB
SP1
IL15
PIK3CD
PRSS1
AGER
LRPPRC
HMGB1
CEL
SLC27A2
MIR23B
LIVAR
FGB
GDF15
SHH
GSR
RARS1
NR1I3
MAOA
SLC10A1
CD163
ALMS1
AHR
NQO1
IL21
AIFM1
HSP90AA1
NDUFA12
UCP3
LITAF
CPS1
RPS27A
DKK1

MIR95
MIR187
GPX1
FTL
NDUFB3
CASP7
NAT2
HBA1
CLCN2
GAL
PLAUR
GFER
NDUFA10
FLI1
NR0B2
DRD1
MIR149
MST1
GARS1
NR5A2
LGALS3
COL3A1
TET2
SHC1
TMPRSS6
ADA2
CCL4
MIR29B1
DNMT3A
CYC1
CD59
EIF2AK4
APPL1
RPS6KB1
POSTN
ABCG1
DNASE1
KL
UQCRC2
IL1RAPL2
USF1
GOLM1
CFLAR
PLTP
NDUFA11
TMPO

EIF4G1
TLR6
PTPN1
NPC1L1
MIR33B
DICER1
PEPD
DGAT2
CYP2B6
STAR
AREG
EPHX1
SERPINF2
TXN
P4HB
UQCRQ
MIR9-1
SDCCAG8
PEMT
FDFT1
CDH2
WNT3A
MERTK
COX8A
TGM2
IL33
HDAC9
PLIN2
KLF6
ANXA2
