# Supplementary figures and images for: Mechanisms of Cynarine for treatment of non-alcoholic fatty liver disease based on the integration of network pharmacology, molecular docking and cell experiment
Source: Hereditas. 2022 Dec 1;159:44. doi: 10.1186/s41065-022-00256-7 (PMC9714250; doi:10.1186/s41065-022-00256-7)

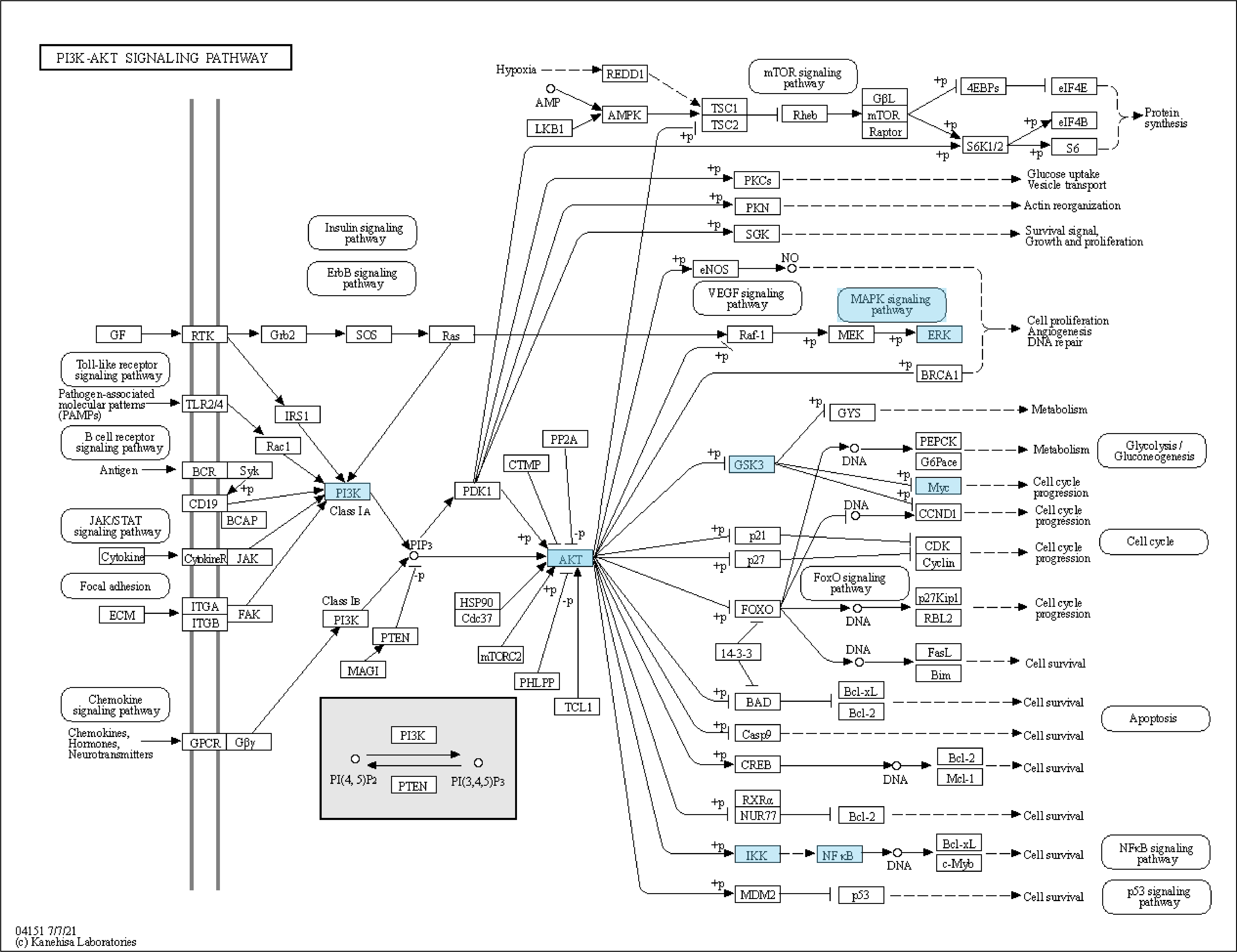

Supplement: Supplementary file 3 — Additional file 3. [file 41065_2022_256_MOESM3_ESM.png]
